# Rare Course of Bilateral Congenital Diaphragmatic Hernia Treated Thoracoscopically—Case Report

**DOI:** 10.3389/fped.2020.00209

**Published:** 2020-04-29

**Authors:** Krystian Toczewski, Sylwester Gerus, Mateusz Palczewski, Dariusz Patkowski

**Affiliations:** Department of Pediatric Surgery and Urology, Wrocław Medical University, Wrocław, Poland

**Keywords:** bilateral congenital diaphragmatic hernia, thoracoscopy, Simpson-Golabi-Behmel syndrome, case report, newborn

## Abstract

We present a rare case of metachronous bilateral congenital diaphragmatic hernia (CDH) in a newborn with additional malformations: macroglossia, pectus excavatum, and confirmed Simpson-Golabi-Behmel syndrome. We performed a successful thoracoscopic subsequent repair with a patch of the bilateral type C CDH. Despite using insufflation that should cause bilateral pneumothorax at first procedure there was no clinical signs of such a one. Contralateral CDH diagnosis was possible only on the basis of the symptoms that occurred after the first operation. After 13 months the patient is without recurrence. In selected patients, staged thoracoscopy may be a safe and feasible method of bilateral CDH treatment.

## Introduction

Congenital diaphragmatic hernia (CDH) is a major birth defect that occurs in ~1 in 2000–3000 live births (1). It is caused by defect in the diaphragm allowing intrathoracic herniation of abdominal viscera what may lead to pulmonary hypoplasia and pulmonary hypertension in consequence (2). Bilateral CDH is a rare form of CDH and accounts for 1–2% of all CDH patients (3). It is often associated with other congenital anomalies and has a higher mortality rate than unilateral CDH (65–74% comparing to 20–35%) (4). It is extremely rare for bilateral CDH to occur as metachronous hernia. We present a first case of successful thoracoscopic staged repair of a bilateral CDH without a sac with untypical clinical course that was not revealed earlier even though the insufflation was used.

## Case Report

A 3,96 kg male neonate was born by cesarean section to a second gravida with gestational diabetes at the 34th week of pregnancy. Family history of genetic diseases was negative. After delivery due to respiratory failure and a poor result of non-invasive ventilation, endotracheal intubation and mechanical ventilation was introduced. An X-ray revealed right CDH with mediastinal shift to the left. On ultrasound examination, a part of the liver was confirmed in the chest. Echocardiography showed hypertrophic cardiomyopathy. Prenatal ultrasound evaluation missed the diagnosis. The patient was transferred to the Pediatric Intensive Care Unit.

After preoperative stabilization on day of life (DOL) 6 a right-sided thoracoscopy was performed. Intermittent CO2 insufflation up to 4–6 mmHg at the beginning and to 2 mmHg after viscera reduction was used. The liver, small and large intestine were identified within the pleural cavity and reduced to the abdominal cavity. No hernia sac was present. The hernia was type C according to the Congenital Diaphragmatic Hernia Study Group classification (3) i.e., over 50% of the diaphragm surface was absent. Because of the size, the defect was repaired with a polypropylene mesh sutured with a combination of intracorporeal interrupted and continuous sutures (braided non-absorbable 2–0 sutures). The patch was attached to the chest wall with percutaneous sutures tied subcutaneously with the PIRS technique used for laparoscopic inguinal hernia repair. Pleural cavity was drained.

Postoperatively the patient was on the high-frequency oscillatory ventilation. Chest radiograph showed a satisfactory result of the right-sided repair. Because of liquid and air leakage the chest was drained for 11 days. 2 days later the newborn was extubated and ventilated with PEEP.

**Figure 1:**
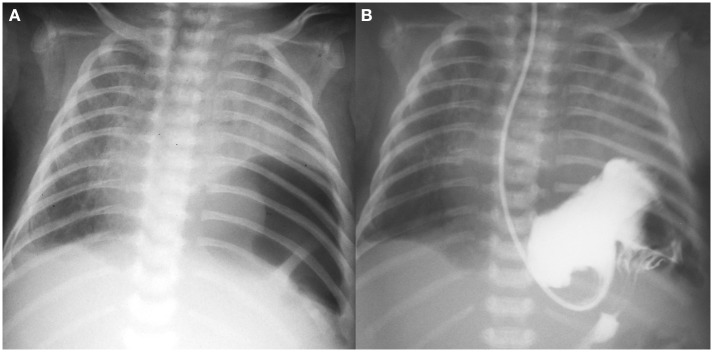
**(A)** Plain chest x-ray with air bubble above the left diaphragm; **(B)** gastric contrast study.

After the next 5 days, the patient presented signs of respiratory distress (labored breathing and diminished breath sound on the left side) with vomiting and excessive gastric fluid. Chest x-ray revealed air bubble above the left diaphragm suggesting diaphragmatic hernia (Figure 1A). The diagnosis was confirmed with gastric contrast study (Figure 1B). On the same day (DOL 25) left-sided thoracoscopy was performed. The diaphragmatic hernia contained stomach and spleen, without any sac. The defect was posterolateral with no rim on the chest wall [type B according to the Congenital Diaphragmatic Hernia Study Group classification (3) i.e., <50% of the diaphragm surface was absent]. After reducing the viscera into the abdominal cavity, it was possible to close the defect without need for a patch. The diaphragm was attached to the chest wall with percutaneous sutures tied subcutaneously (the PIRS technique used for laparoscopic inguinal hernia repair) and the second layer of interrupted sutures (braided non-absorbable 2-0). A chest drain was placed. Chest x-ray on the postoperative day 3 confirmed acceptable lung expansion and the drain was removed. On DOL 29 the patient was extubated and became NIV and oxygen independent on DOL 35.

Both procedures of thoracoscopic repair despite using the CO2 insufflation were tolerated well by the patient without significant changes in ventilation during the operations.

On 13 months follow-up the patient remains without CDH recurrence. However, the patient developed pectus excavatum and macroglossia. Because of feeding difficulties, the reduction glossectomy has been planned. Due to abovementioned malformations and coarse facies the patient had genetic testing and diagnosis of Simpson-Golabi-Behmel syndrome was established.

## Discussion

Bilateral CDH is uncommon (around 1–2% of all CDH) and unusual clinical course with late metachronous onset on the other side makes it an extremely rare entity. We have managed to find just two described cases of metachronous bilateral CDH but both patients had a hernia sac at least on one side (5, 6). To our best knowledge this is the first case of bilateral CDH without a sac with metachronous presentation.

The most frequent clinical presentation of CDH is cardiopulmonary distress with cyanosis, tachypnoea, and tachycardia. Gastrointestinal symptoms are also common, especially in the left-sided CDH (3, 7). In our patient first respiratory and, after right-sided repair, gastrointestinal and respiratory signs were present. In bilateral CDH there is a connection between both pleural cavities through the abdomen. Hence after thoracoscopic one-side repair contralateral pneumothorax should be expected. Interestingly in our case after right-sided thoracoscopic repair there was no intraoperative clinical signs as well as the postoperative radiograph shows no left-side pneumothorax. Either the contralateral pneumothorax was effectively drained with the chest tube or the viscera were tightly closing the diaphragmatic defect on the left side preventing the gas to enter contralateral pleural space.

Video-assisted thoracic repair of CDH was first described by Silen et al. (8). Since then thoracoscopic treatment has been gaining wider acceptance (9). However, many believe that because of CO2 used for insufflation, the thoracoscopic technique would be harmful to patients with CDH. Our patient tolerated thoracoscopy very well. Moreover, it is worth underlining that due to the nature of the disease the operative space in the chest after viscera reposition combined with small hypoplastic lungs make the insufflation almost unnecessary therefore it can be lowered to minimal values. To our knowledge, this is the first thoracoscopic treatment of a bilateral CDH with at least one side patch repair in the world literature and the second successful bilateral thoracoscopic repair described whatsoever (5). Thoracoscopic patch repair carries a higher risk of recurrence comparing to open and primary repairs (10). We, like others, believe that patch failure is strongly related to the technique of implantation (1). The size and shape of the patch were estimated using threads cut to measure the diaphragm defect size in two dimensions. The patch should be carefully anchored to the thoracic wall and the diaphragm edges in a loose dome-shaped fashion without tension (10, 11). Using non-absorbable sutures and proper operating technique (including thick tissue bites on the chest wall) may lower the risk of recurrence even in minimally invasive patch repair. CDH itself, patch repair as well as Simpson-Golabi-Behmel syndrome all predispose to pectus excavatum. Whether the chest deformity is related to the type of reconstruction or to the defect size remains unclear (11).

## Conclusion

Bilateral CDH is not always diagnosed straight away. In newborns after unilateral CDH repair with a sudden respiratory distress or gastrointestinal signs a contralateral CDH should be considered in the differential diagnosis. Contralateral CDH might not be visible in radiographs before one-side repair. Although chest x-ray is usually sufficient to confirm the diagnosis, in ambiguous cases a contrast study may be helpful.

In selected cases, thoracoscopic repair is possible in metachronous bilateral CDH and may be taken into consideration as a treatment method even in large defects requiring patch repair.

## Data Availability Statement

The datasets generated for this study are available on request to the corresponding author.

## Ethics Statement

Written informed consent was obtained from the minor(s)' legal guardian/next of kin for the publication of any potentially identifiable images or data included in this article.

## Author Contributions

SG devised the project and provided critical feedback. KT wrote the manuscript. MP collected material and prepared the Figures. DP helped with the discussion and supervised the project. All authors discussed and contributed to the final manuscript.

## Conflict of Interest

The authors declare that the research was conducted in the absence of any commercial or financial relationships that could be construed as a potential conflict of interest.
